# Screening low-methanol and high-aroma produced yeasts for cider fermentation by transcriptive characterization

**DOI:** 10.3389/fmicb.2022.1042613

**Published:** 2022-11-11

**Authors:** Liang Liu, Peng Tao Zhao, Ching Yuan Hu, Dan Tian, Hong Deng, Yong Hong Meng

**Affiliations:** ^1^The Engineering Research Center for High-Valued Utilization of Fruit Resources in Western China, Ministry of Education, National Research and Development Center of Apple Processing Technology, College of Food Engineering and Nutritional Science, Shaanxi Normal University, Xian, China; ^2^Department of Human Nutrition, Food and Animal Sciences, College of Tropical Agriculture and Human Resources, Honolulu, HI, United States

**Keywords:** *Saccharomyces cerevisiae*, *Pichia bruneiensis*, cider, methanol, alcohols, esters

## Abstract

The commercial active dry yeast strains used for cider production in China are far behind the requirements of the cider industry development in recent decades. In this study, eight yeasts, including *Saccharomyces cerevisiae*, *Schizosaccharomyces pombe*, *Pichia bruneiensis*, and *Pichia kudriavzevii*, were screened and assessed by growth performance, methanol production, aroma analysis, and their transcriptive characterization. *Saccharomyces cerevisiae* strains WFC-SC-071 and WFC-SC-072 were identified as promising alternatives for cider production. Strains WFC-SC-071 and WFC-SC-072 showed an excellent growth capacity characterized by 91.6 and 88.8% sugar utilization, respectively. Methanol production by both strains was below 200 mg/L. Key aroma compounds imparting cider appreciably characteristic aroma increased in cider fermented by strains WFC-SC-071 and WFC-SC-072. RT-qPCR analysis suggested that most genes associated with growth capacity, carbohydrate uptake, and aroma production were upregulated in WFC-SC-071 and WFC-SC-072. Overall, two *Saccharomyces cerevisiae* strains are the optimal starters for cider production to enable the diversification of cider, satisfy the differences in consumer demand, and promote cider industry development.

## Introduction

Cider, with an alcohol content of 1.2–8.5%, is the second most-consumed fruit wine globally (Lorenzini et al., 2019; Zhang et al., 2021). As a fruit beverage, apple cider has the characteristics of both apple juice and beer, such as being golden in color, rich in fermented aroma, strong palatability, and mellow mouthfeel. Moreover, cider has nutrients from apples and fermentation products; thus, it has a higher nutritional value (Lee et al., 2019; Zhang et al., 2020; Nie et al., 2021). As a result, cider is becoming a promising product, especially in the eastern Asian markets (Khandpur and Gogate, 2015; Guine et al., 2021). Due to the rapid expansion of the cider market, developing and producing high-quality cider is a priority for the cider industry.

Yeasts play a decisive role in fermentation and eventually determine cider quality. They are the primary contributor to the color, aroma, product features, and styles of ciders (Song et al., 2020). In addition, yeast converts sugars and other substrates to ethanol, carbon dioxide, and other aromatic substances during fermentation (Fejzullahu et al., 2021). Traditional ciders are produced through natural fermentation by autochthonous yeasts. However, the number of wild microbes in mature fruit pericarps and stems far exceeds autochthonous yeasts. These wild microbes often interfere with yeast fermentation, thus affecting the quality of fruit wine (Lorenzini et al., 2019). Moreover, commercial active dry yeast strains are ubiquitously used for winemaking, but little research on unique strains for cider fermentation. Therefore, it is necessary and significant to identify appropriate yeasts for cider fermentation.

Various yeasts have been isolated from different fruits and fermented grains to endow fruit wines with high concentrations of pleasant aromas and distinctive floral fragrances in winemaking (Callejon et al., 2010; Saberi et al., 2012). *Saccharomyces cerevisiae*, isolated from a vineyard in Piedmont (Italy), was used to produce wine with an alcohol content of 13.29 g/L, as well as a pleasant smell of rose (Costantini et al., 2021). *Saccharomyces cerevisiae GP-34* was isolated from the natural fermentation liquid of loquat juice. Compared with commercial *Saccharomyces cerevisiae*, the contents of alcohols, esters, acids, and phenols in loquat wine fermented by *Saccharomyces cerevisiae GP-34* were 31, 86, 25, and 11% higher, respectively (Xu et al., 2019).

Meanwhile, the pleasant flavor of fruit wine is mainly attributed to non-*Saccharomyces* yeasts, which shape the chemical profile of aromatic compounds and enhance the diversity of aroma (van Wyk et al., 2019; Godoy et al., 2020). Several non-*Saccharomyces* yeasts have been identified and utilized to confer characteristic flavors to fruit wines. For example, Torulaspora delbrueckii, obtained from Brazilian culture collection, synthesized large amounts of phenethyl acetate during fermentation, thereby conferring a fruity fragrance (Van Mullem et al., 2022). However, data on the promising yeast for cider processing are still scarce, and new strains shall be identified to generate diversified styles of ciders.

Moreover, methanol, a hazard factor of fruit wine fermentation, is derived from the methyl group of pectin hydrated by yeast enzymes (Wu et al., 2017). Methanol is toxic and can cause weakness, vomiting, coma or even death when intaken excessively (Padilla et al., 2016; Ghosh et al., 2021). Consequently, it is critical to comprehensively evaluate the strains and obtain dominant strains capable of improving overall cider quality and diversifying cider styles to meet the needs of a wide range of consumers.

In the present study, 75 yeast strains were isolated from the apples harvested in Changwu, Shaanxi Province, China’s renowned hometown of apples, and fermented grains from Maotai and Xifeng, two well-known Chinese liquors. A simple olfactory test was used to screen the isolated strains, and eight yeasts producing desirable aromas were obtained. Their identities were confirmed by 28S rRNA analysis. These eight yeast strains were then used for cider-making to evaluate their growth capacity, methanol production, and volatile production. Based on these assessments, two yeast strains were identified as promising alternatives for cider fermentation. In summary, this work promotes the diversification of cider, potentially satisfies the differences in consumer demand, and promotes cider industry development.

## Materials and methods

### Materials

Fresh Red Fuji apples (56.7% fructose, 10.4% glucose, and 32.9% sucrose) were purchased from Changwu, Shaanxi province, China. The apples were kept in cold storage before subsequent studies. The fermented grains were obtained from Maotai (Guizhou) and Xifeng Distilleries (Shaanxi), China. All organic solvents were chromatographic grade and purchased from Sigma-Aldrich (Shanghai, China). Total RNA extractor reagent (B511311) was obtained from Sangon Biotech (Shanghai, China). One-step gDNA Removal kit (AT341-02) and Perfectstart™ Green qPCR SuperMix kit (AQ601-02) were purchased from TransGen Biotech (Beijing, China).

### Enrichment and isolation of yeast strains

Apples were soaked in 75% ethanol for 5 min, washed twice with sterile water, then dipped in 2% sodium hypochlorite for 5 min, washed four times with sterile water, and cut into 1 cm^3^ squares. Five grams of the fermented grains obtained from Maotai and Xifeng Distilleries and 5 g of apple squares were mixed with 100 ml yeast peptone glucose (YPD; 2% glucose, 2% peptone, and 1% yeast extract) at 30°C for 24 h. Then, culture fluids enriched with yeasts were spread separately over YPD agar and incubated at 30°C for 48 h. Next, 75 yeast colonies were selected and streaked on YPD agar plates for purification. This process was repeated in triplicate (Kumari et al., 2020). Finally, each strain was coded and stored at −80°C for subsequent experiments.

### Fermentation trial

Apple juice was prepared from peeled apples using a juicer (Hurom, Korea; model HUF8800STS). Vitamin C (1‰) was added to inhibit apple juice oxidation and discoloration. Fresh apple juice was sterilized at 100°C for 30 s before inoculation. All strains obtained in section “Enrichment and isolation of yeast strains” were used to ferment apple juice. Each yeast strain was pre-cultured in 50 ml YPD medium at 30°C for 1 day on a rotary shaker with a speed of 200 rpm, separated by centrifugation (5,000 × *g*, 4°C, 5 min), washed twice with sterile water, and inoculated in 200 ml sterilized apple juice for cider production at a concentration of approximately 10^7^ CFU/ml. The fermentations were conducted at 30°C for 7 days.

### Screening and identification of yeast strains

Twenty students and 10 staff from Shaanxi Normal University were recruited as screeners. They performed a simple olfactory test to describe and grade cider aroma, and strains with a low score and undesirable aroma were excluded (Gutiérrez et al., 2018). Eight strains were selected using this screening test. The specific scoring form and scoring guidelines are shown in Supplementary Table S1.

The eight strains selected by the olfactory test were further identified. These yeasts were inoculated in YPD at 30°C for 1 day. Cell morphology was observed with an optical microscope (Nikon, Japan; model ECLIPSE E100). These strains were identified by 28S rRNA. The genomic DNA of yeast was extracted by the SDS-based method (Xia et al., 2019). The D1/D2 domain of the 28S rRNA was amplified with primers NL1 and NL4 (Mikhailova et al., 2020). The sequences were then compared with those in the GenBank and analyzed using BLAST software. Phylogenetic tree construction of these yeasts was performed using MEGA X software.

### Assessment of yeast strains

#### Sugar utilization

Total sugar content of cider was determined using the phenol−sulfuric acid method. Sample preparation was conducted using a modified existing method (Wu et al., 2022). Briefly, 100 μl of cider was diluted with 1 ml of ultrapure water, and then mixed with 1 ml of 5% phenol and 5 ml of concentrated sulfuric acid. The mixture was incubated in a water bath at 35°C for 30 min. The sample absorbance was measured at 490 nm using a UV–VIS Spectrophotometer (Optima, Japan). A glucose standard solution is prepared as a reference solution. The standard linear equation generated was y = 0.0061x−0.0073, *r*^2^ = 0.998.

#### Methanol production

Methanol content in cider was analyzed using an existing method with modifications (Arslan et al., 2015). The cider was distilled to collect the volatile components and filtered through a 0.25 μm membrane filter (Jinlong, China). Methanol was analyzed using a GCMS-QP2010SE system (Shimadzu, Japan) coupled with a flame ionization detector (FID; Shimadzu, Japan) and a DB-5 capillary column (2 m × 2 mm I.d.; film thickness: 1.5 μm; Chrompack, Netherlands). The oven temperature started at 40°C for 3 min, and increased to 100°C at a rate of 5°C/min, then increased to 200°C at 20°C/min and maintained for 5 min. The single injection analysis time was 25 min. The FID temperature was set at 250°C. Detector flow rates of 20, 400, and 25 ml/min were applied to hydrogen, air, and helium (make-up). A column flow rate of 1 ml/min was applied to helium (as carrier gas). Each sample was measured in triplicate. Methanol was identified according to retention time and quantified *via* the external standard method.

### Aroma analysis

#### Quantitative descriptive analysis

Quantitative descriptive analysis (QDA) was conducted by 10 evaluators (five males and five females). The evaluation was based on a modified procedure (Zhao et al., 2021). All assessors were trained to be familiar with cider aroma assessment. They were instructed to assess the ciders in eight aroma attributes: acid, sweet, fruity, alcoholic, spicy, smooth, dry, and dense. Five milliliter of cider were transferred in a 20 ml sterile cup, marked with a random number, and scored using a scale of 0–9 (0 = none, 9 = very strong). Sensory evaluation of each sample was carried out in triplicate and at room temperature, and the results for each aroma attribute were averaged and plotted on a radar chart.

#### Analysis of aroma compounds by HS-SPME-GC-MS

Extraction of aroma compounds in cider was performed using a headspace solid-phase micro-extraction (HS-SPME) according to an existing method with minor modifications (Niu et al., 2019). In brief, 5 ml cider, 2 g sodium chloride, and 10 μl 3-octanol (10 mg/L, internal standard) were placed in a 20 ml micro-reaction vial. The vial was put into a thermostatic water bath at 70°C for 30 min. The 50/30 μm divinylbenzene/carboxen/polydimethylsiloxane (DVB/CAR/PDMS; Sigma-Aldrich, United States; 57348-U) fiber was exposed to the sample headspace (about 1 cm above the liquid surface) at 70°C for 30 min, injected in the GC injector and kept for 3 min, and analyzed using an Agilent 8890 GC system equipped with a 5977B mass spectrometer (MS) according to a modified method (Niu et al., 2019). Chromatographic separations were performed using a DB-wax column (20 m × 0.18 mm, Agilent, Santa Clara, CA, United States). The column flow rate of helium was 1.5 ml/min. Electron energy of 40 Ev and mass range of m/z 30–450 were applied during MS analysis. The initial temperature was maintained at 40°C for 6 min, increased to 100°C at 4°C /min rate, then increased to 230°C at 10°C/min rate, and held for 20 min. Each sample was measured in triplicate.

### RT-qPCR

Total cellular RNA was extracted using Trizol reagent (Sangon Biotech, China; B511311-0100) according to the manufacturer’s instructions. Samples were quantified using a Nanophotometer N60 Touch spectrophotometer (Implen GmbH, Germany; T60765). The reverse transcription reaction system was prepared according to the manufacturer’s protocol, containing 1 μg of the RNA sample. Primer sequence, reaction efficiency and threshold were described in Supplementary Table S2. Real-time PCR was performed using a Perfectstart™ Green qPCR SuperMix kit. Amplification parameters include an initial denaturation stage at 95°C for 3 min, followed by 40 cycles of 10 s at 95°C and 63°C for 30 s, and a final extension stage at 72°C for 5 min. A dissociation protocol was carried out to verify the non-specific amplification absence. The mRNA abundance change was calculated using the 2[-Delta Delta C(T)] method (Lorca Mandujano et al., 2022). Beta-actin (*ACT1*) gene was used as the internal control gene.

### Data analysis

Origin 2021 software (Origin Lab Co., Northampton, Massachusetts, United States) was used for graphing. All experiments were conducted in triplicates, and results were expressed as means ± SD. One-way ANOVA, followed by Duncan’s multiple range test, was performed using the SPSS 21.0 software (SPSS Inc., Chicago, IL, United States). The difference between the means was considered significant when *p* < 0.05.

## Results

### Eight yeast strains with pleasant aroma were identified from different sources

Yeasts from apples and fermented grains were cultured in an YPD medium, and 75 yeast strains were obtained. We performed a simple olfactory test described in section “Screening and identification of yeast strains” of the Materials and Methods to screen aroma-producing yeasts rapidly. We selected eight isolates based on their high scores during the olfactory test. Furthermore, these eight strains also endowed ciders with predominantly fruity or floral flavors or a combination of fruit and floral flavors. Thus, these eight strains (WFC-014, 045, 047, 048, 051, 054, 071, and 072) were selected for subsequent molecular analysis (Table 1).

**Table 1:** Aroma-producing characteristics and sensory analysis of fermented ciders from 75 different yeasts.

Source	Strain	Odor descriptions	Color	Scores	Source	Strain	Odor descriptions	Color	Scores
Apple	1	Dull, sweet	Tawny	4	Maotai wine fermented grains	39	Dull, sweet	Tawny	3
	2	Dull, sweet	Brownish black	4		40	Dull, sweet	Tawny	3
	3	Alcohol, sweet	Tawny	6		41	Delicate	Brownish black	2
	4	Stink	Brownish black	1		42	Weak alcohol	Tawny	6
	5	Dull	Brownish black	1		43	Fruity	Tawny	5
	6	Dull, sweet	Tawny	4		44	Delicate	Tawny	3
	7	Stink	Brownish black	1		45	Weak alcohol, fruity	Pale yellow	8
	8	Sweet	Tawny	3		46	Delicate		2
	9	Acid smell	Brownish black	2		47	Fruity, flower, alcohol	Pale yellow	9
	10	Dull, sweet	Brownish black	2		48	Ripe sweet, weak alcohol	Pale yellow	8
	11	Dull, sweet	Brownish black	2		49	Stink	Tawny	1
	12	Delicate	Brownish black	2		50	Stink	Tawny	1
	13	delicate	Brownish black	2		51	Strong alcohol, fruity	Pale yellow	8
	14	Alcohol, strong fruity	Pale yellow	8		52	Delicate	Tawny	2
	15	Acid smell	Brownish black	2	Xifeng wine fermented grains	53	Delicate	Brownish black	2
	16	Dull, sweet	Brownish black	2		54	Weak alcohol, fruity	Pale yellow	8
	17	Acid smell	Brownish black	2		55	Weak alcohol	Tawny	6
	18	Acid smell	Brownish black	2		56	Acid smell	Brownish black	1
	19	Delicate	Brownish black	2		57	Acid smell	Brownish black	1
	20	Delicate	Brownish black	2		58	Acid smell	Brownish black	1
	21	Dull, sweet	Brownish black	2		59	Acid smell	Brownish black	1
	22	Acid smell	Brownish black	2		60	Strong fruity	Tawny	6
	23	Delicate	Brownish black	2		61	Stink	Brownish black	1
	24	Flower	Tawny	6		62	Delicate	Brownish black	2
	25	Alcohol	Tawny	6		63	Acid smell	Brownish black	1
	26	Delicate	Brownish black	2		64	Acid smell	Brownish black	1
	27	Sweet	Brownish black	3		65	Delicate	Brownish black	1
	28	Fruity	Tawny	6		66	Delicate	Brownish black	1
	29	Hawthorn taste	Tawny	6		67	Acid smell	Brownish black	1
	30	Dull, sweet	Tawny	4		68	Acid smell	Brownish black	1
	31	Dull, sweet	Tawny	4		69	Acid smell	Brownish black	1
	32	Dull, sweet	Tawny	4		70	Strong alcohol	Tawny	6
	33	Dull, sweet	Tawny	4		71	Alcohol, fruity	Pale yellow	9
	34	Delicate	Brownish black	2		72	Strong alcohol, fruity	Pale yellow	9
	35	Delicate	Brownish black	2		73	Weak alcohol, stink	Brownish black	1
	36	Delicate	Brownish black	2		74	Weak alcohol, stink	Brownish black	1
	37	Delicate	Brownish black	2		75	Acid smell	Brownish black	1
	38	Delicate	Brownish black	2					

**Figure 1:**
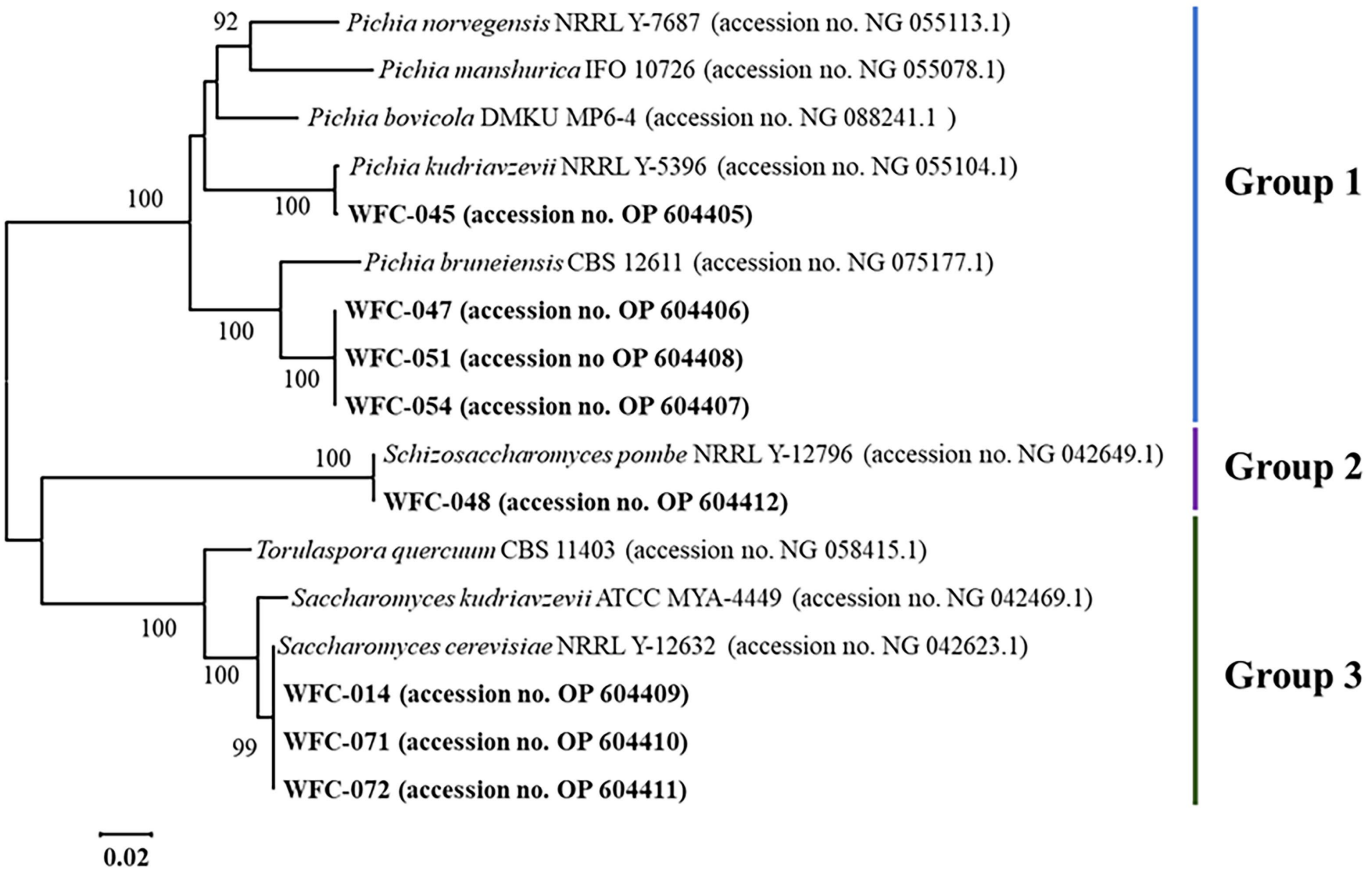
Phylogenetic trees of 28S rRNA of yeasts.

The phylogenetic tree of the yeast species was established by MEGA X software based on 28S rRNA sequences (Figure 1). The type of selected strains fell into three distinct groups. Four strains of group 1 clustered with *Pichia* type (accession no. NG 075177.1 and NG 055104.1) and shared a clade. Strain WFC-045 (accession no. OP 604405) was more closely related to *Pichia kudriavzevii* NRRL Y-5396, as the bootstrap value between them was 100. And the three remaining strains were very close to *Pichia bruneiensis* CBS 12611. A strain of group 2 clustered with the *Schizosaccharomyces* type (accession no. NG 042649.1) and was very close to *Schizosaccharomyces pombe* NRRL Y-12796. At the same time, three strains of group 3 clustered with *Saccharomyces cerevisiae* type (accession no. NG 042623) and were very close to *Saccharomyces cerevisiae* NRRL Y-12632. The four *Pichia* yeasts of group 1 were named WFC-PK-045, WFC-PB-047, WFC-PB-051, and WFC-PB-054. The *Schizosaccharomyces pombe* strain of group 2 was named WFC-SP-048. And the three *S. cerevisiae* strains of group 3 were named WFC-SC-014, WFC-SC-071, and WFC-SC-072.

### Assessment of the fermentation performance of eight yeasts

**Figure 2:**
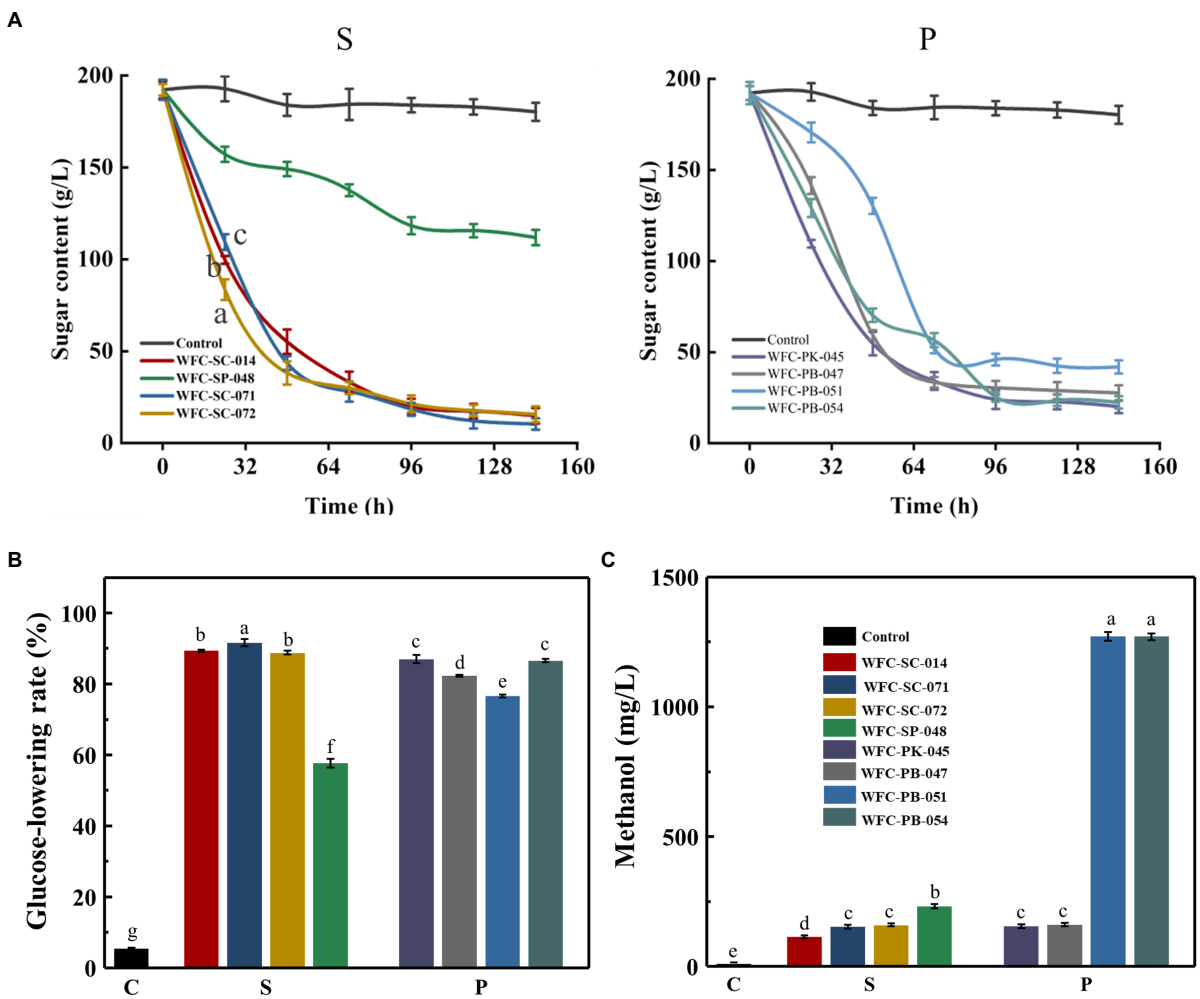
Growth capability of isolated strains on apple juice. **(A)** Total sugar consumption by *Saccharomyces cerevisiae* strains and *Schizosaccharomyces pombe* strain (S), and *Pichia* yeasts (P). **(B)** Glucose-lowering rate of apple juice fermented by different strains for 7 days. Means within glucose-lowering rate between different yeasts with different letters (a–g) differ significantly (*p* < 0.05). Both sugar consumption and glucose-lowering rate experiments were replicated three times. **(C)** Methanol concentration in ciders fermented by eight yeasts. Means with different letters (a–e) differ significantly (*p* < 0.05). Most strains had robust growth capability and produced acceptable amounts of methanol.

One of the crucial characteristics of yeast suitable for cider production is its growth ability (Wang et al., 2022). Yeasts use carbon sources (mainly sugar) to ferment and produce high-sensory quality cider (Lorca Mandujano et al., 2022). Therefore, sugar utilization was evaluated to determine the yeast fermentation capacity of these eight yeast strains. The three *S. cerevisiae* strains showed a similar sugar utilization tendency during the whole fermentation (Figure 2A). Nevertheless, the sugar consumption rate (0–24 h) and total sugar consumption were significantly different (*p* < 0.05; Figures 2A,B). Strain WFC-SC-072 showed a maximum sugar consumption rate (0–24 h), whereas strain WFC-SC-071 exhibited the highest total sugar consumption. Typically, ethanol accumulation inhibits non-*Saccharomyces* yeasts’ metabolism, resulting in less sugar utilization (Bao et al., 2021). However, strains WFC-PK-045, WFC-PB-047, and WFC-PB-054 consumed sugar by up to 80% (Figure 2B). The sugar consumption level by strain WFC-PK-045 was close to the *S. cerevisiae* strains. Strain WFC-SP-048 was the only strain with lower sugar consumption capability. These results indicate that most strains had robust growth capability.

Methanol content is a critical parameter of cider quality. Its content should be strictly controlled below 400 mg/L (Wei et al., 2020b). Therefore, these yeast strains’ methanol production capacity was measured (Figure 2C). Compared to the *Saccharomyces cerevisiae* yeasts, *Pichia* yeast strains produced more methanol. Among all *Pichia* yeast strains, WFC-PB-051 and WFC-PB-054 had the highest methanol (1,270 mg/L). Most strains produced little methanol, below 300 mg/L. No difference (*p* > 0.05) in methanol production was observed among *Pichia* yeasts WFC-PK-045 and WFC-PB-047, *Saccharomyces* strains WFC-SC-071 and WFC-SC-072. Notably, *Saccharomyces* strain WFC-SC-014’s methanol production (114.2 mg/L) was the lowest of all strains and lower than commercial *Saccharomyces cerevisiae* (Chen et al., 2019). In summary, six strains with methanol content below 400 mg/L could be used for cider production.

### Sensory evaluation and quantitative descriptive analysis of ciders fermented by eight yeasts

**Figure 3:**
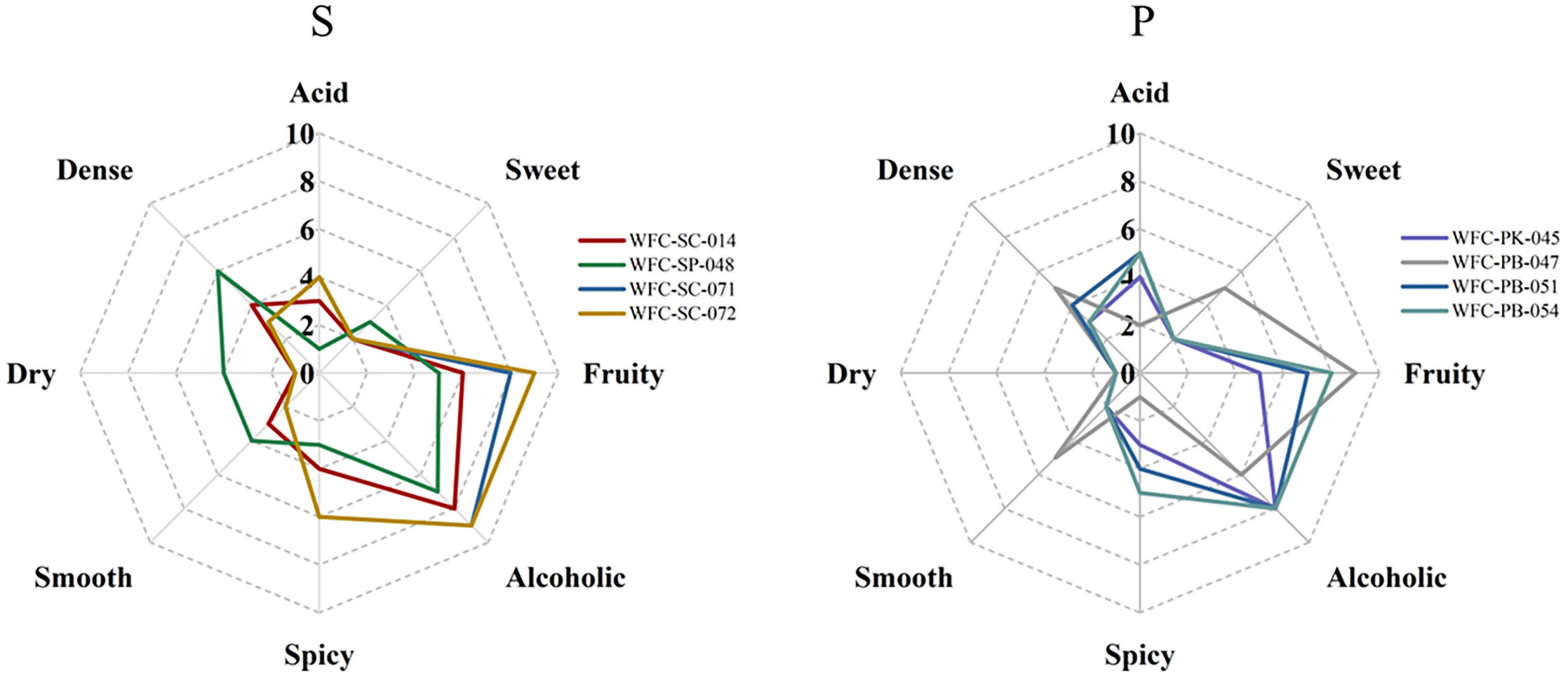
Sensory descriptive analysis of ciders fermented by *Saccharomyces cerevisiae* strains and *Schizosaccharomyces pombe* strain (S) or *Pichia* yeasts (P). Cider fermented by *Saccharomyces cerevisiae* strains WFC-SC-071, WFC-SC-072, or *Pichia* yeast WFC-PB-047 has desirable aroma characteristics for the cider style.

Quantitative descriptive analysis (QDA) was performed to evaluate the aroma traits under different yeast fermentation conditions (Figure 3). Eight aroma attributes, acid, sweet, fruity, alcoholic, spicy, smooth, dry, and dense, were representative of odor attributes. A least significant difference (LSD) analysis shows that “dense,” “acid,” “spicy,” and “fruity” attributes differed among Group S and the “fruity” attribute differed among Group P (*p* < 0.05). As for the overall aroma profile, fruity and alcohol attributes scores were higher than the other attributes, suggesting that they were the predominant aroma attributes in cider. Regarding the fruity attribute, it was the maximum intensity perceived in cider, which could be mainly interpreted by the synthesis of acetates and fatty acid ethyl esters during fermentation. In addition, the cider fermented by strain WFC-SC-071 exhibited a similar profile compared to that cider fermented by strain WFC-SC-072, with both being similar to commercial ciders. Overall, these results indicate that *Saccharomyces cerevisiae* strains WFC-SC-071, WFC-SC-072, and *Pichia* yeast WFC-PB-047 resulted in valuable and desirable characteristics of the cider style.

### SPME-GC–MS analysis of volatile composition profiles of ciders

Aroma and flavor define cider quality, while molecule profiles account for organoleptic properties (Qin et al., 2018). Cider aroma compounds mainly comprise esters, alcohols, fatty acids, acetyls, terpenes, and other trace components (Nespor et al., 2019). The aromatic profiles were drastically improved after fermentation.

**Figure 4:**
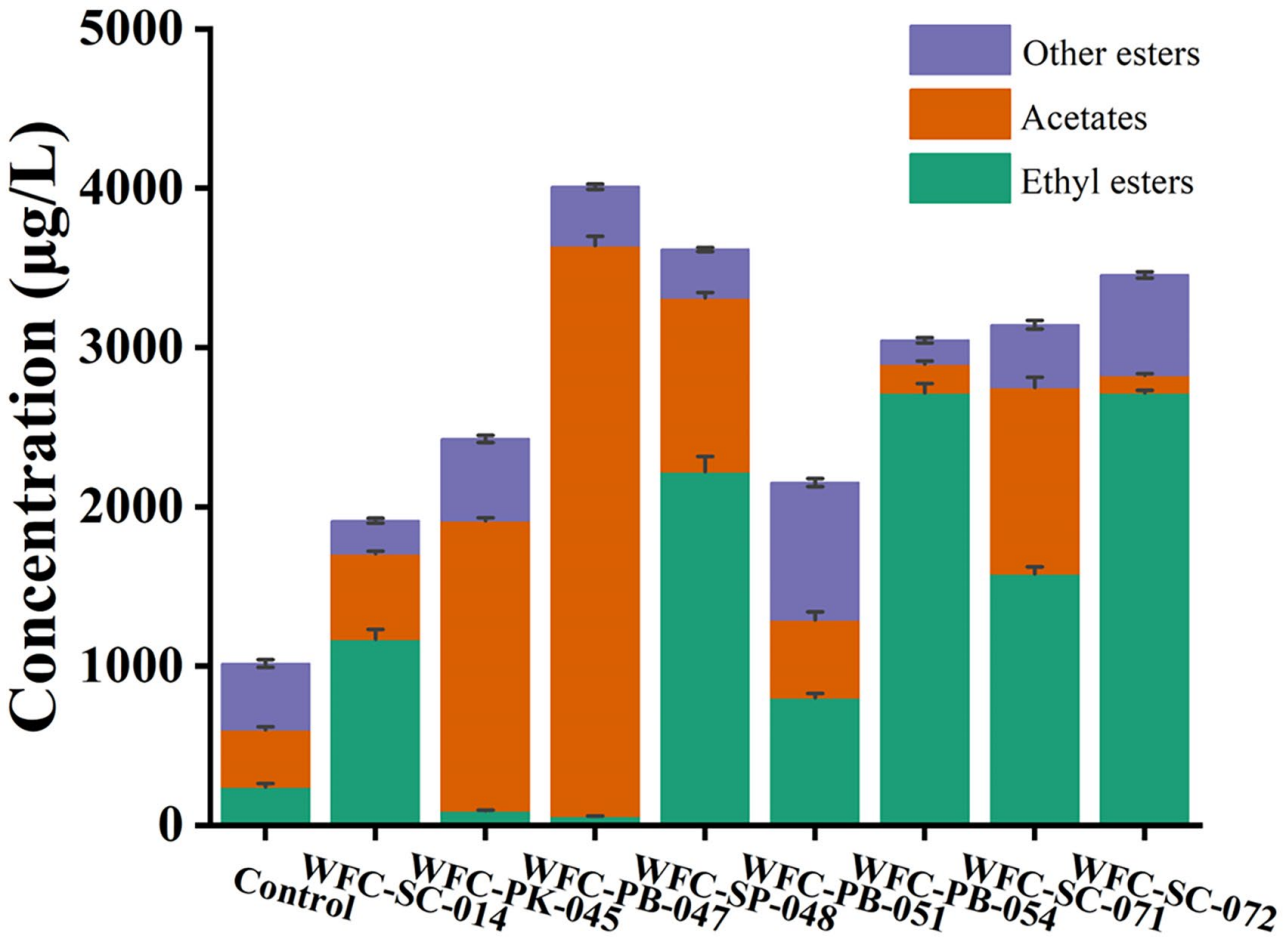
Ester production in ciders fermented by different yeasts. The orange bar indicates average acetate concentration, the green bar represents ethyl esters, and the purple bar shows other esters. All eight strains had outstanding ester production capacity to improve cider sensory quality.

Esters are ciders’ predominant group of volatile compounds (Aung et al., 2015). Esters are synthesized *via* esterification during fermentation; they have a fruity odor. For fruity esters, fermentation improved ester production (Figure 4). Fermentation by these eight yeasts improved esters abundance, especially acetates and fatty acid ethyl esters. As shown in Supplementary Table S3, fermentation by all eight strains elevated acetates and fatty acid ethyl esters species to various degrees. Ester abundance of cider fermented by strain WFC-SC-071 or WFC-PB-047 resulted in four or five times higher than the unfermented group. In addition, esters were significantly increased (*p* < 0.05) after fermentation by these eight strains. The total ester content of cider fermented by strain WFC-PB-047 was about four times more than the unfermented group. Typically, acetate in WFC-PB-047 was about 10 times more than in the unfermented group.

**Figure 5:**
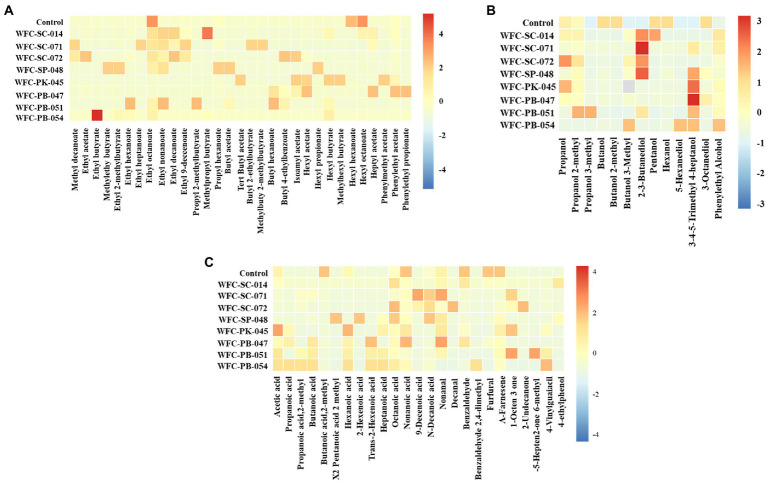
Volatile compounds of the fermented ciders. The heat map describes the aromatic compounds of ciders fermented for 7 days by eight strains. Esters **(A)**, alcohols **(B)**, and other aroma compounds **(C)** in fermented ciders. Aroma components changed significantly after fermentation. Aroma compounds were prominently expressed in ciders fermented by strains WFC-SC-071 and WFC-SC-072.

Ciders fermented by different yeast strains showed contrasting ester profiles (Figure 5A). For example, the content of 11 aroma compounds in ciders fermented by strains WFC-SC-071 and WFC-SC-072 were significantly higher (*p* < 0.05) than in unfermented apple juice. Similarly, seven compounds were accumulated in cider fermented by strain WFC-PB-047. Ethyl esters and acetates, among esters, are the most determinant compounds contributing to cider flavor, such as ethyl acetate, ethyl octanoate, ethyl nonanoate, and isoamyl acetate. Ethyl nonanoate was detected in ciders fermented by strains WFC-SC-014, WFC-SC-071, WFC-SC-072, WFC-SP-048, and WFC-PB-051, imparting ciders’ grape and rose flavor. Ethyl octanoate, with pineapple and sweet flavor, was detected before and after fermentation, although the abundance changed significantly (*p* < 0.05). However, hexyl hexanoate and hexyl octanoate decreased by 74–100% (*p* < 0.01) after fermentation (Supplementary Table S3).

Alcohols were the second largest group among the volatile compounds in ciders. Most alcohols are produced from sugar and amino acid catabolism during fermentation (Qin et al., 2018). The alcohol content in apple juice decreased significantly after fermentation (Supplementary Table S3). However, many key alcohols imparting cider appreciably characteristic aroma increased and became an essential component in the resultant ciders (Figure 5B). More than 10 alcohols were highly expressed in several ciders, excluding cider fermented by strain WFC-SC-014. Among alcohols, 2, 3-butanediol, 3-methyl-1-butanol, and phenylethyl alcohol are pivotal substances contributing to cider style. Phenylethyl alcohol, which has honey, spicy, rose, and lilac aromas, was concentrated in all ciders compared to apple juice. 3-Methyl-1-butanol was found most bountiful in ciders fermented by strains WFC-SC-014, WFC-SC-072, WFC-PB-047, and WFC-PB-054, and this compound contributes to a whiskey odor in cider even in low concentration. However, the relative abundance of 1-butanol and 2-methyl-1-butanol was reduced after fermentation.

Acids are mainly produced during fermentation (Wei et al., 2020a). As shown in Figure 5C, ciders contained various volatile acids, while the unfermented apple juice only had a considerable level of 2-methyl butanoic acid. High increments of short-chain fatty acids were observed in *Pichia* yeast strains, while *Saccharomyces cerevisiae* yeast strains generated large amounts of medium-chain fatty acids. The aldehydes are generated *via* alcohol oxidation or acid decarboxylation during fermentation (Xiao et al., 2015). Several aldehydes, including benzaldehyde, nonanal, and decanal, were identified in ciders. Nonanal, a crucial aromatic compound in fruit wines was enriched in seven ciders after fermentation. With almond and burnt sugar odor, benzaldehyde was suggested to foster characteristic sensory properties (Sun et al., 2016). An abundance of benzaldehyde was detected in ciders fermented by strains WFC-SC-014 and WFC-PB-047. Moreover, 1-octen-3-one, a volatile unsaturated ketone with a strong mushroom-like odor, was enriched in four ciders. 6-Methyl-5-hepten-2-one, which has a strong citrus-like odor, was abundant in cider made by strain WFC-PB-051. Extraordinary, phenolic off-flavors like 4-vinylguaiacil and 4-ethylphenol, which have a negative impact on the overall aromatic profile, were detected in cider fermented by strains WFC-PK-045 and WFC-PB-047 (Alvarez Gaona et al., 2021). In conclusion, aromatic profiles were drastically improved after fermentation. The ideal aroma profile was presented in ciders fermented by strains WFC-SC-071 and WFC-SC-072.

### Analysis of eight strains’ gene expression during fermentation

**Figure 6:**
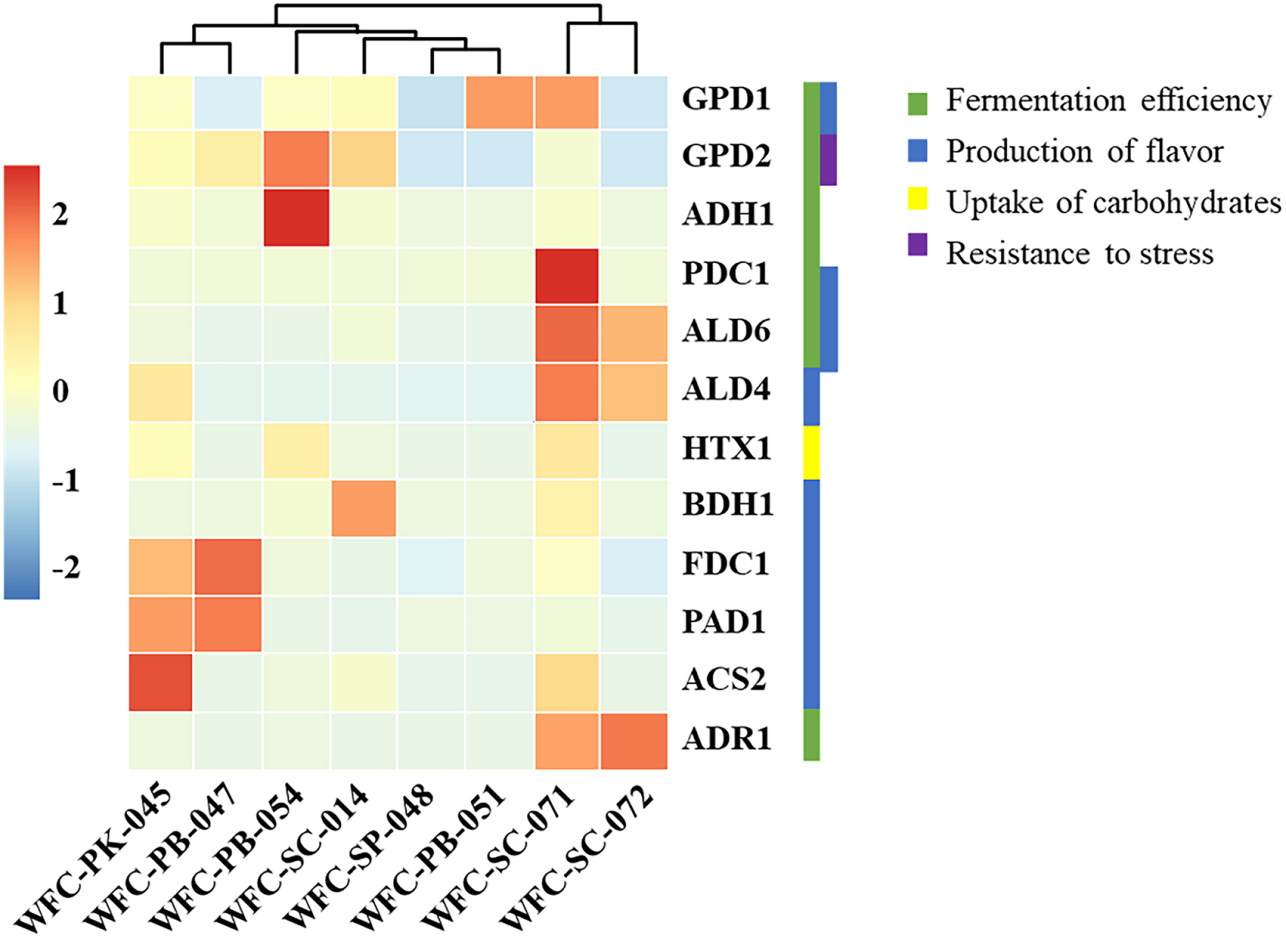
Changes in gene expression. The results are expressed as the median of the difference in gene expression level before and after the fermentation of triplicate samples. Genes in green, blue, yellow, and purple are related to fermentation capacity, flavor production, carbohydrate uptake, and stress resistance, respectively. Strains WFC-SC-071 and WFC-SC-072 demonstrated ideal expression levels of most genes associated with aroma production, carbohydrate uptake, and yeast fermentation capacity.

Different yeast strains possess different genetic predispositions, thus profoundly influencing fermentation. RNA samples were collected both before and after fermentation to evaluate the transcription level changes of the yeasts during fermentation. Using RT-qPCR, we quantitatively analyzed the gene expression levels related to anaerobic stress resistance, aroma production, carbohydrate uptake, and yeast growth capacity to investigate the characteristic yeast properties. Gene expression levels are presented in Figure 6, and the average values are provided in Supplementary Table S4.

Gene expression analysis revealed deviated metabolic pathways during fermentation. *GPD1* and *GPD2*, two homologous genes, regulated NAD-dependent glycerol-3-phosphate dehydrogenase (Gpd1p and Gpd2p), act on dihydroxyacetone phosphate to form glycerol-3-phosphate (Vilela, 2021). *GPD1* is related to glycerol production during osmotic conditions (Marullo and Dubourdieu, 2022). *GPD2* is associated with modulating the balance of NADH-NAD redox under osmotic stress (Sun et al., 2022). *GPD1* and *GPD2* expression is specifically upregulated in response to anaerobic stress (Baeza et al., 2022). Among the eight strains, both strains WFC-PB-051 and WFC-SC-071 showed higher expression levels of *GPD1*, while strain WFC-PB-054 exhibited more pronounced upregulation of *GPD2* (*p* < 0.05). The upregulated expression of *GPD1* and *GPD2* levels in strains WFC-PB-051, WFC-PB-054, and WFC-PB-047 might be interpreted as adapting to the anaerobic environment during fermentation.

Alcohol dehydrogenase I, encoded by *ADH1*, reduces acetaldehyde to ethanol during fermentation (Raj et al., 2014). *ADH1* was upregulated in all eight strains, and comparatively higher expression levels were measured in strain WFC-PB-054. *PDC1* gene encoding an isoform of pyruvate decarboxylase was activated among eight strains, and higher expression levels were detected in strain WFC-SC-071. *PDC1* is related to the non-oxidative conversion of pyruvate to acetaldehyde and carbon dioxide during fermentation and participates in amino acid catabolism (Dzialo et al., 2017). Yeasts lacking *PDC1* activity grow slowly for an inadequate acetyl CoA supply or an imbalanced cytoplasmic redox (Mitsui et al., 2022).

Expression levels of *ADR1*, *ALD4*, and *ALD6* were prominent in WFC-SC-071 and WFC-SC-072. *ADR1*, encoding a transcription activator of glucose-repressible alcohol dehydrogenase, was repressed in the absence of glucose, while its activation occurs upon glucose exhaustion (Zhang G. et al., 2022). *ALD6*, encoding the cytosolic acetaldehyde dehydrogenase, is indispensable for converting acetaldehyde to acetate (Wang et al., 2020). These genes are also intercorrelated as *ADR1* activates genes related to glucose fermentation, including *ALD4* and *ALD6* (Kalender and Çalık, 2020)*.* The expression levels of these genes associated with the respiratory cycle, uptake and metabolism of carbohydrates exhibited an upregulated metabolic profile in strains WFC-SC-071 and WFC-SC-072 during fermentation.

*PAD1* and *FDC1*, participating in the decarboxylation of aromatic compounds and carboxylic acids generated during fermentation, were markedly activated in the WFC-PK-045 and WFC-PB-047 strains. Like *ADR1* and *ALD4*, the expression of *PAD1* and *FDC1* are also interdependent. Together, the two genes are related to the production of the clove-like off-flavor during fermentation. In addition, *PAD1*, encoding flavin prenyltransferase, is involved in the decarboxylation of phenylacrylic acids, such as ferulic acid, *p*-coumaric acid, and cinnamic acid, producing the corresponding vinyl derivatives (Gulmezian et al., 2007; Lin et al., 2015).

Interestingly, the expression levels of *ACS2* were prominently upregulated in strains WFC-SC-071 and WFC-PK-045. Among these genes, *ACS2* encodes an acetyl-CoA synthetase and may contribute to ethanol tolerance and ethyl-acetate synthesis of yeast cells (Xu et al., 2021a,b). *BDH1* gene is overexpressed in strain WFC-SC-014 and is responsible for reducing acetoin to 2,3-butanediol, a neutral-scented compound (Ehsani et al., 2009).

The *HXT1* gene encodes carriers for hexose transport and plays as the sugar transporter during yeast growth, specialized for glucose and fructose (Munoz-Miranda et al., 2022). Strains in the absence of *HXT1* genes could not grow on glucose or fructose and have no glycolytic flux (Nadai et al., 2021). *HXT1* was activated in strains WFC-PK-045, WFC-PB-054, and WFC-SC-071 and exhibited lower expression levels at the end of fermentation.

Strains WFC-SC-071, WFC-SC-072, WFC-PB-047, and WFC-PB-054 exhibited upregulated transcription profiles for most genes associated with aroma production, carbohydrate uptake, and yeast fermentation capacity. The upregulated transcription profiles verified that strains WFC-SC-071 and WFC-SC-072 possess desirable fermentation and aroma properties.

## Discussion

Cider is the second most-consumed fruit drink and is becoming a promising drinking product (Cousin et al., 2017). Urgent requirements for diverse and innovative ciders have emerged. Aroma and flavor formed by yeast metabolism inarguably define cider organoleptic quality and consumer acceptance (Bingman et al., 2022). Therefore, it is necessary to identify new yeast strains for cider fermentation to enhance the aroma compound profile. In this study, we identified two *Saccharomyces cerevisiae* with excellent growth capacity, capable of generating an abundant aroma compound profile. They are selected as promising starters for cider production.

### Alcohols

Higher alcohols, with fruity and floral odors, are one of the most integral traits that define the unique aroma profiles of cider (Hinkley et al., 2022). Higher alcohols are synthesized from amino acids *via* the anabolic pathway or the catabolic (Ehrlich) pathway (Wang et al., 2019). The Ehrlich pathway is a three-step and pivotal biochemical pathway in aroma production. Strains WFC-SC-071 and WFC-SC-072 increased the key higher alcohol content during fermentation, such as phenethyl alcohol. This higher alcohol accumulation in ciders may be attributed to the upregulation of *PDC*1 and *BDH1*, as these genes are responsible for reducing aldehydes to alcohols (Varga et al., 2021). At the same time, 3-methyl butanol content in strain WFC-SC-071 was lower than strains WFC-SC-072 and WFC-PB-047 after fermentation, owing to the low expression level of *ALD6* in strain WFC-SC-071. The higher expression level of *ALD6* will result in increased acids and reduced alcohols, such as isobutanol (Zheng et al., 2020). Moreover, plenty of 2,3-butanediol was measured in ciders fermented by strains WFC-SC-071 and WFC-SC-014, which may transform from acetoin (a buttery-like smell), by the *BDH1* overexpression in strains WFC-SC-071 and WFC-SC-014 (Bae et al., 2021). Notably, *GPD1* and *GPD2* upregulation in strain WFC-SC-071 may tune ethanol and glycerol content to produce cider with lower ethanol content (Vicente et al., 2021).

Methanol is typically detected in ciders and derives from the methyl group of pectin hydrated *via* yeast enzymes (Pressman et al., 2020). Methanol can also be synthesized *via* the yeast glycine metabolic pathway. Glycine produces methylamine by the action of yeast glycine decarboxylase, which reacts with nitrite to produce methanol (Liang et al., 2015). *In vivo* accumulation of methanol will cause blindness, coma, and metabolic disturbances that incur high mortality (Botelho et al., 2020). It has been previously reported that the methanol content of cider fermented by commercial *Saccharomyces cerevisiae* Fermicru LS2, Zymaflore VL1 and RM1515 was, respectively, 225, 227, and 219 mg/L (Nicolini et al., 2018). Methanol in ciders fermented by the two yeasts selected, 148.9 and 155.8 mg/L, were comparatively lower than commercial yeasts. Methanol content in ciders is closely associated with enzymatic activities during fermentation. Therefore, less methanol in the selected yeasts may be attributed to lower pectin methyl esterase and glycine decarboxylase expression (Blumenthal et al., 2021).

Moreover, *Pichia* yeasts, categorized as methylotrophic yeasts, rely on methanol or their unique methanol utilization pathways to grow. Therefore, *Pichia bruneiensis* WFC-PB-047 can induce less methanol accumulation than *Saccharomyces cerevisiae*. Since they produce less methanol, these two selected yeasts possess the potential for cider production. Nevertheless, lower methanol (25 mg/L) has been observed in cider preprocessed with bentonite and malic in apple juice before fermentation (Bedrinana et al., 2019; Simonato et al., 2021). Therefore, additional treatment could be conducted in subsequent fermentation trials to further reduce methanol production.

### Esters

Esters are a vast and cardinal group of compounds in ciders. They confer pleasant fruity and floral aroma nuances to cider, thus improving perceptual quality (Munoz-Gonzalez et al., 2020). The total ester content in cider fermented by commercial *Saccharomyces cerevisiae* S288C was 2,077 mg/L (Zhang Z. et al., 2022). In this study, the total ester in the two selected yeasts was 3,455 and 3,672 mg/L, respectively, significantly exceeding commercial *Saccharomyces cerevisiae*. Acetates and fatty acid ethyl esters, the most determinant compounds contributing to the fruity and sweet scent, were dramatically increased in diversity and abundance, with their species increasing from 2 to 9 and 3 to 11, respectively (Simonato et al., 2021). Acetate is derived from pyruvate, the terminal product during glycolysis and plays a central role in carbon metabolism. Pyruvate converts to acetate *via* two pathways, namely coupling with reactive oxygen species (ROS) or catalyzed by ketoacid dehydrogenases, which have a neomorphic function as pyruvate decarboxylases (Liu et al., 2018). Acetate synthesis relies on catalysis by pyruvate decarboxylase (PDC; Mews et al., 2017; Bradshaw, 2021). Acetaldehyde dehydrogenase (ALD) is also essential in cytosolic acetyl-CoA and lipid production (Koivuranta et al., 2018). High expression of *PDC1*, *ALD4*, and *ALD6* in the selected yeasts explained the increase in both abundance and species of acetates. Ethyl octanoate, with pineapple and candy flavor, was detected at 0.13 mg/L in Edinburgh Yeast, 0.16 mg/L in Belgian II Yeast, 0.29 mg/L in French Saison Yeast, and 0.2 mg/L in Abbey Yeast (Bingman et al., 2020), whereas, 126.9 mg/L in the WFC-PB-047 evaluated herein. Ethyl nonanoate, absent in apple juice, was highly expressed in the two selected yeasts after fermentation. Ethyl esters, as secondary metabolites in yeasts during fermentation, are synthesized upon ethanol consumption (Knight et al., 2014). Ethanol is generated from acetaldehyde by yeast alcohol dehydrogenase I encoded by the *ADH1* gene (Raj et al., 2014). Upregulated expression of *PDC1*, which is also associated with ethanol production, supplementarily contributes to ciders’ ethanol level (Dickinson et al., 2003). Therefore, the accumulation of fatty acid ethyl esters may be associated with the high expression of *ADH1* and *PDC1* in the two selected yeasts.

In general, these selected strains positively enhanced apple juice fermentation and improved cider quality in multiple aspects. Additionally, some strains with slight flaws but agreeable overall performance, characterized by inferior cider product quality, could be enhanced and compensated by co-culturing with the other strains of complementary characters. This work promotes the diversification of cider, satisfies the differences in consumer demand, and will promote cider industry development in the future.

## Data availability statement

The datasets presented in this study can be found in online repositories. The names of the repository/repositories and accession number(s) can be found in the article/Supplementary material.

## Author contributions

LL: conceptualization, data curation, formal analysis, writing–original draft, and visualization. PZ: resources, supervision, writing–reviewing and editing, and methodology. CH: writing–reviewing and editing. DT: data curation. HD: supervision and project administration. YM: resources, project administration, writing–reviewing and editing, supervision, and funding acquisition. All authors contributed to the article and approved the submitted version.

## Funding

This research was supported by the National Natural Science Foundation of China (31972089) and the 2020 Team Innovation Project from the Fundamental Scientific Research Special Capital Fund of the National Universities, China (GK202001008).

## Conflict of interest

The authors declare that the research was conducted in the absence of any commercial or financial relationships that could be construed as a potential conflict of interest.

## Publisher’s note

All claims expressed in this article are solely those of the authors and do not necessarily represent those of their affiliated organizations, or those of the publisher, the editors and the reviewers. Any product that may be evaluated in this article, or claim that may be made by its manufacturer, is not guaranteed or endorsed by the publisher.

## Supplementary material

The Supplementary material for this article can be found online at: https://www.frontiersin.org/articles/10.3389/fmicb.2022.1042613/full#supplementary-material

SUPPLEMENTARY TABLE 1 The scoring form and guidelines.

SUPPLEMENTARY TABLE 2 Sequence of primers used in gene expression analyses.

SUPPLEMENTARY TABLE 3 Volatile compounds of ciders produced from apple juice fermentation carried out by eight strains. “-” indicates no detection.

SUPPLEMENTARY TABLE 4 Values of gene expression used in heat map analysis. The values are a media of triplicates.
