# Supplementary material for: Screening low-methanol and high-aroma produced yeasts for cider fermentation by transcriptive characterization
Source: Front Microbiol. 2022 Nov 11;13:1042613. doi: 10.3389/fmicb.2022.1042613 (PMC9691974; doi:10.3389/fmicb.2022.1042613)
Supplement: SUPPLEMENTARY TABLE 1 — The scoring form and guidelines. [file Table_1.DOCX]

**Supplementary table S1:**

| scores (0 - 10) | Sweetness | Acidity | Odor | Color |
| --- | --- | --- | --- | --- |
| 7-10 | Moderate | Moderate | Desirable | Pale yellow |
| 4-6 | Strong | Strong | Harmonious | Tawny |
| 1-3 | Extreme | Extreme | Stink & dull | Brownish black |
