# Supplementary material for: Screening low-methanol and high-aroma produced yeasts for cider fermentation by transcriptive characterization
Source: Front Microbiol. 2022 Nov 11;13:1042613. doi: 10.3389/fmicb.2022.1042613 (PMC9691974; doi:10.3389/fmicb.2022.1042613)
Supplement: SUPPLEMENTARY TABLE 2 — Sequence of primers used in gene expression analyses. [file Table_2.DOCX]

**Supplementary table S2: Sequence of primers used in gene expression analyses**

| **Gene** | **Primer sequence** | **Threshold** | **Efficiency (%)** |
| --- | --- | --- | --- |
| **GPD1** | FW: CATTGCCACCGAAGTCGCTC | 0.1 | 98 |
|  | RV: GCCCTCGCCTCTGAAATCCT |  |  |
| **GPD2** | FW: TTCGAGTTGGGCTCCAAGGG | 0.1 | 100 |
|  | RV: ACCAATGCTCCTTGGCCACT |  |  |
| **ADH1** | FW: CGGTGCTGTTCTAAAGGCCAC | 0.111 | 96 |
|  | RV: GCATACCGACCAAAACGGTGG |  |  |
| **PDC1** | FW: CGCCGCTAAGGGTTACAAGC | 0.22 | 104 |
|  | RV: TAGAAGCTGGGACAGCAGCG |  |  |
| **ALD6** | FW: CCTTAGCCCGTGGGGATGTT | 0.097 | 95 |
|  | RV: GCCGTCACCGGTGTTGATTG |  |  |
| **ALD4** | FW: GCGGACGCCGAGTTGAAAAA | 0.109 | 98 |
|  | RV: TGAACCCGCACAACAGACCT |  |  |
| **BDH1** | FW: GGGGTCCAAAACCTGTCCCA | 0.26 | 103 |
|  | RV: TGTCTCCGTTGTGGATGGCA |  |  |
| **ACS2** | FW: TGGTTCTGCTACCGTGCCAT | 0.099 | 96 |
|  | RV: ACGGTCGTGGTGGTTCCAAA |  |  |
| **HTX1** | FW: GCTGGCAGAATCGACGAAGC | 0.7 | 100 |
|  | RV: GCAGTACCAGCGGCTCTCAT |  |  |
| **FDC1** | FW: TTAGGTTGCCCAGCCGGTTT | 0.07 | 100 |
|  | RV: GAGCCCCAGATGATGGGCAA |  |  |
| **PAD1** | FW: TCCTCCGGTACCTGCGTTTT | 0.072 | 99 |
|  | RV: AGTGTCAGCGTGGATGCCAA |  |  |
| **ADR1** | FW: ACGAGAGCGTTCGCAAGACA | 0.072 | 100 |
|  | RV: GTTGCAGAGGCCACAGGGAT |  |  |
