# Supplementary material for: Screening low-methanol and high-aroma produced yeasts for cider fermentation by transcriptive characterization
Source: Front Microbiol. 2022 Nov 11;13:1042613. doi: 10.3389/fmicb.2022.1042613 (PMC9691974; doi:10.3389/fmicb.2022.1042613)
Supplement: SUPPLEMENTARY TABLE 3 — Volatile compounds of ciders produced from apple juice fermentation carried out by eight strains. “-” indicates no detection. [file Table_3.DOCX]

**Supplementary table S3:** **Volatile compounds of ciders produced from apple juice fermentation carried out by eight strains. “-”** **indicates no detection.**

| **Concentration (μg/L)** | **control** | **WFC-SC-014** | **WFC-PK-045** | **WFC-PB-047** | **WFC-SP-048** | **WFC-PB-051** | **WFC-PB-054** | **WFC-SC-071** | **WFC-SC-072** |
| --- | --- | --- | --- | --- | --- | --- | --- | --- | --- |
| **1-Propanol** | **79.9±2.92** | **14.1±0.58** | **45.8±0.95** | **12.5±0.65** | **3.1±0.29** | **10.4±0.34** | **1.6±0.05** | **3.8±0.22** | **112.2±6.1** |
| **1-Propanol,2-methyl-** | **86.6±3.15** | **18.3±0.62** | **22.3±0.88** | **14.5±1.09** | **11.7±0.24** | **154.2±5.09** | - | **12.9±0.74** | **86.6±4.71** |
| **1-Propanol,3-methyl-** | - | - | - | - | - | **226.3±7.47** | - | - | - |
| **1-Butanol** | **1854.1±42.88** | **16.5±0.57** | **47.1±1.84** | **35.2±0.6** | **32.8±1.08** | **54.8±1.81** | **100.4±3.28** | **19.2±1.1** | **40.9±2.22** |
| **1-Butanol,2-methyl-** | **3584.2±234.4** | **2.5±0.25** | **2.2±0.58** | - | - | **3.0±0.13** | **8.1±0.26** | **0.1±0.01** | - |
| **1-Butanol,3-Methyl** | - | **215.8±7.42** | - | **122.3±7.1** | **140.2±4.48** | - | **2987.6±97.6** | - | **1085.8±59.3** |
| **2,3-Butanediol** | - | **8.3±0.41** | - | - | **8.5±0.59** | **16.7±0.55** | - | **26.9±1.54** | **26.8±1.46** |
| **1-Pentanol** | **2002.3±109.4** | **561.6±14.9** | **1.1±0.21** | **1.4±0.21** | **1.6±0.22** | **4.8±0.16** | **8.3±0.27** | **2.2±0.13** | **1.6±0.09** |
| **1-Hexanol** | **2677.6±135.1** | **16.8±0.63** | **22.8±0.95** | **23.1±0.99** | **61.3±2.73** | **181.4±5.99** | **153.1±5.0** | **44.3±2.54** | **28.0±1.52** |
| **1,5-Hexanediol** | - | - | - | - | - | - | **195.1±6.37** | - | - |
| **3,4,5-Trimethyl-4-heptanol** | - | - | **17.9±0.82** | **22.9±1.6** | **7.3±0.62** | **29.3±0.97** | **31.3±1.02** | **9.2±0.53** | - |
| **1,3-Octanediol** | **986.6±6.36** | - | **33.8±1.05** | **198.5±9.19** | **69.2±23.79** | **96.7±3.19** | **16.3±0.53** | - | **18.1±0.98** |
| **Phenylethyl Alcohol** | **456.9±24.76** | **504.7±5.81** | **445.3±8.06** | **318.4±9.64** | **119.6±4.34** | **1159.5±38.3** | **3049.5±99.6** | **825.0±47.3** | **445.5±24.2** |
| **Total alcohols** | **11728.2** | **1358.6** | **638.3** | **748.8** | **455.3** | **1937.1** | **6551.3** | **943.64** | **1845.5** |

| **Concentration (μg/L)** | **control** | **WFC-SC-014** | **WFC-PK-045** | **WFC-PB-047** | **WFC-SP-048** | **WFC-PB-051** | **WFC-PB-054** | **WFC-SC-071** | **WFC-SC-072** |
| --- | --- | --- | --- | --- | --- | --- | --- | --- | --- |
| **Methyl decanoate** | **-** | **-** | **18.7±1.3** | **11.3±1.2** | **-** | **-** | **-** | **-** | **-** |
| **Ethyl Acetate** | **-** | **17.4±2.1** | **13.5±2.1** | **487.6±7.9** | **-** | **0.8±0.02** | **20.6±2.07** | **-** | **-** |
| **Ethyl butyrate** | **-** | **17.2±3.1** | **13.0±2.9** | **-** | **5.7±0.87** | **-** | **5.7±0.17** | **-** | **3206.5±65** |
| **2-Methylethyl butyrate** | **2.4±0.3** | **-** | **-** | **-** | **243.8±4.76** | **-** | **-** | **-** | **-** |
| **Ethyl 2-methylbutyrate** | **-** | **2.4±0.1** | **14.8±3.1** | **-** | **168.3±3.21** | **-** | **-** | **-** | **21.4±2.17** |
| **Ethyl hexanoate** | **-** | **3.3±0.2** | **2.0±0.9** | **15.6±1.3** | **4.6±0.97** | **-** | **-** | **97.3±4.12** | **10.6±1.07** |
| **Ethyl heptanoate** | **-** | **-** | **64.7±7.31** | **-** | **-** | **-** | **-** | **-** | **-** |
| **Ethyl octanoate** | **184.5±5.7** | **89.1±3.7** | **142.5±9.7** | **126.9±4.7** | **90.6±2.34** | **3.7±0.85** | **4.9±0.76** | **68.0±2.74** | **34.4±3.31** |
| **Ethyl nonanoate** | **-** | **4.8±0.7** | **7.4±1.1** | **3.8±0.9** | **6.5±1.01** | **-** | **-** | **8.3±1.01** | **-** |
| **Ethyl decanoate** | **-** | **972.1±8.7** | **712.3±7.1** | **1665.1±9.9** | **-** | **-** | **20.5±2.51** | **70.8±3.74** | **-** |
| **Ethyl 9-deccenoate** | **18.5±1.08** | **76.6±7.1** | **647.2±6.7** | **396.1±7.2** | **9.6±1.27** | **-** | **-** | **7.8±1.07** | **13.7±1.21** |
| **Propyl 2-methylbutyrate** | **-** | **0.3±0.01** | **-** | **-** | **-** | **-** | **3.7±0.72** | **148.5±5.12** | **-** |
| **2-Methylpropyl butyrate** | **-** | **98.0±3.2** | **-** | **0.4±0.09** | **-** | **-** | **0.1±0.08** | **-** | **5.7±0.86** |
| **Propyl hexanoate** | **76.5±3.17** | **-** | **22.4±1.2** | **-** | **102.0±3.21** | **8.2±1.71** | **4.2±0.58** | **3.2±0.37** | **0.9±0.04** |
| **Butyl acetate** | **-** | **2.1±0.07** | **0.3±0.1** | **13.9±1.42** | **866.4±11.3** | **0.9±0.07** | **8.1±1.21** | **259.7±6.72** | **9.3±4.12** |
| **tert-Butyl acetate** | **-** | **-** | **-** | **-** | **-** | **150.4±5.12** | **-** | **-** | **-** |
| **Butyl 2-methylbutyrate** | **-** | **1.0±0.03** | **1240.9±12** | **-** | **-** | **2.1±0.76** | **0.4±0.01** | **3.2±0.84** | **20.6±2.12** |
| **2-Methylbutyl 2-methylbutyrate** | **-** | **-** | **86.0±4.45** | **-** | **-** | **-** | **-** | **-** | **-** |
| **Butyl hexanoate** | **-** | **0.1±0.01** | **-** | **-** | **5.3±0.21** | **-** | **155.9±5.71** | **228.7±7.91** | **-** |
| **2-Butyl 4-ethylbenzoate** | **-** | **-** | **-** | **83.7±5.37** | **-** | **-** | **15.5±2.17** | **30.8±4.62** | **-** |
| **Isoamyl acetate** | **-** | **-** | **-** | **75.0±7.21** | **-** | **79.2±3.17** | **30.8±3.71** | **-** | **-** |
| **Hexyl acetate** | **-** | **4.5±0.08** | **1.6±0.21** | **19.3±2.17** | **0.9±0.07** | **161.5±4.01** | **171.0±4.12** | **1.4±0.43** | **-** |
| **Hexyl propionate** | **-** | **1.5±0.12** | **0.4±0.03** | **0.4±0.03** | **165.8±7.21** | **0.8±0.02** | **1.8±0.21** | **3.0±1.20** | **-** |
| **Hexyl butyrate** | **-** | **29.0±1.2** | **-** | **-** | **-** | **133.1±3.17** | **-** | **40.9±4.12** | **26.0±1.72** |
| **2-Methylhexyl butyrate** | **-** | **-** | **-** | **1.7±0.12** | **-** | **346.4±4.97** | **-** | **-** | **-** |
| **Hexyl hexanoate** | **135.9±4.21** | **-** | **-** | **-** | **0.7±0.07** | **2.6±0.37** | **4.9±0.98** | **-** | **-** |
| **Hexyl octanoate** | **180.1±6.37** | **47.0±1.4** | **-** | **-** | **4.5±0.97** | **-** | **0.1±0.04** | **1.1±0.21** | **2.8±0.53** |
| **Heptyl acetate** | **-** | **28.5±1.9** | **39.0±1.27** | **-** | **-** | **-** | **106.2±4.21** | **0.6±0.02** | **-** |
| **2-Phenylmethyl acetate** | **-** | **-** | **-** | **-** | **-** | **124.8±3.81** | **-** | **-** | **-** |
| **2-Phenylethyl acetate** | **305.4±5.7** | **469.6±12.7** | **546.8±9.76** | **415.2±7.8** | **226.0±5.2** | **1282.±11.7** | **3265.9±34** | **126.1±4.12** | **-** |
| **2-Phenylethyl propionate** | **-** | **-** | **16.5±2.15** | **-** | **-** | **-** | **180.3±4.21** | **-** | **-** |
| **Total esters** | **634.0821** | **2036.317** | **2452.733** | **5229.495** | **2483.612** | **21176.28** | **1340.854** | **6146.889** | **2861.575** |

| **Concentration (μg/L)** | **Control** | **WFC-SC-014** | **WFC-PK-045** | **WFC-PB-047** | **WFC-SP-048** | **WFC-PB-051** | **WFC-PB-054** | **WFC-SC-071** | **WFC-SC-072** |
| --- | --- | --- | --- | --- | --- | --- | --- | --- | --- |
| **Acetic acid** | **154.7±3.14** | **22.6±1.21** | **99.4±7.21** | **32.4±2.18** | **24.4±2.14** | **186.0±3.12** | **323.8±6.17** | **20.5±1.02** | **10±0.84** |
| **Propanoic acid** | **-** | **-** | **1.5±0.08** | **2.2±0.51** | **-** | **-** | **14.1±1.01** | **-** | **-** |
| **Propanoic acid,2-methyl-** | **-** | **-** | **-** | **-** | **3.4±0.54** | **63.5±2.17** | **189.5±2.61** | **28.0±0.43** | **28.4±0.31** |
| **Butanoic acid** | **-** | **-** | **-** | **10.7±1.21** | **4.9±0.71** | **28.6±1.21** | **57.7±1.07** | **9.1±0.07** | **-** |
| **Butanoic acid, 2-methyl-** | **3048.2±53.1** | **22.6±3.21** | **65.3±1.21** | **84.4±4.72** | **63.0±1.32** | **178.9±5.21** | **469.4±7.21** | **123.4±1.07** | **90.9±1.23** |
| **2-Pentanoic acid,2-methyl-** | **-** | **-** | **-** | **-** | **0.4±0.07** | **-** | **-** | **-** | **-** |
| **Hexanoic acid** | **147.8±2.17** | **1.5±0.12** | **105.2±4.15** | **54.3±3.17** | **-** | **186.1±6.21** | **388.9±6.51** | **-** | **3.8±0.02** |
| **2-Hexenoic acid** | **-** | **-** | **-** | **-** | **2.3±0.15** | **-** | **-** | **-** | **-** |
| **trans-2-Hexenoic acid** | **-** | **-** | **-** | **20.1±1.63** | **-** | **32.4±1.24** | **68.6±1.21** | **-** | **-** |
| **Heptanoic acid** | **-** | **-** | **3.5±0.21** | **-** | **7.2±0.47** | **12.8±0.97** | **26.9±0.96** | **-** | **-** |
| **Octanoic acid** | **364.1±7.27** | **525.8±9.17** | **124.3±3.71** | **151.2±5.71** | **1351.3±37.2** | **360.5±4.27** | **1094.2±23.2** | **163.3±2.16** | **1404.6±13.2** |
| **Nonanoic acid** | **136.2±2.17** | **9.9±0.98** | **22.2±1.21** | **41.6±3.21** | **37.1±1.32** | **21.9±1.07** | **69.3±2.14** | **30.6±1.37** | **29.9±2.71** |
| **9-Decenoic acid** | **-** | **-** | **6.0±0.98** | **-** | **-** | **11.9±0.87** | **42.3±1.27** | **586.7±9.86** | **247.9±6.71** |
| **n-Decanoic acid** | **223.4±4.57** | **-** | **19.2±1.07** | **29.1±1.32** | **1318.9±46.2** | **132.2±2.14** | **342.2±7.31** | **1009.0±45.1** | **1022.2±37.9** |
| **Nonanal** | **9.6±0.92** | **3.1±0.54** | **3.2±0.21** | **6.6±0.96** | **12.2±0.97** | **7.2±0.75** | **-** | **19.3±0.98** | **3.4±0.41** |
| **Decanal** | **-** | **-** | **-** | **-** | **-** | **-** | **-** | **-** | **2.2±0.23** |
| **Benzaldehyde** | **57.6±0.76** | **16.7±0.78** | **3.3±0.47** | **11.1±0.75** | **4.5±0.54** | **-** | **7.1±0.23** | **4.4±0.24** | **5.3±0.51** |
| **Benzaldehyde,2,4-dimethyl-** | **-** | **-** | **-** | **-** | **-** | **-** | **24.6±1.21** | **-** | **-** |
| **Furfural** | **524.8±12.43** | **27.7±1.01** | **3.1±0.21** | **24.1±0.74** | **3.9±0.24** | **1.8±0.21** | **10.6±0,98** | **11.6±0.98** | **3.4±0.41** |
| **a-Farnesene** | **116.0±4.21** | **4.0±0.27** | **15.2±1.17** | **9.8±0.98** | **36.9±1.37** | **35.4±1.24** | **42.1±1.23** | **5.9±0.42** | **15.5±1.09** |
| **1-Octen-3-one** | **-** | **-** | **0.2±0.02** | **-** | **-** | **0.7±0.04** | **-** | **0.5±0.02** | **0.2±0.05** |
| **2-Undecanone** | **-** | **-** | **0.5±0.07** | **-** | **0.9±0.04** | **0.7±0.01** | **0.3±0.02** | **1.4±0.07** | **24.4±1.21** |
| **5-Hepten2-one,6-methyl-** | **-** | **-** | **-** | **-** | **-** | **4.1±0.12** | **-** | **-** | **-** |
| **4-****Vinylguaiacil** |  |  | **30.26±1.21** | **42.28±2.17** |  | **3.61±0.14** | **-** | **-** | **-** |
| **4-ethylphenol** |  | **9.53±0.12** |  |  | **8.51±0.52** |  |  |  |  |
| **other compounds** | **5416.5** | **1115.534** | **979.96** | **3391.2** | **4144.5** | **4439.9** | **5185.4** | **4905.8** | **2892.1** |
