# Supplementary material for: Screening low-methanol and high-aroma produced yeasts for cider fermentation by transcriptive characterization
Source: Front Microbiol. 2022 Nov 11;13:1042613. doi: 10.3389/fmicb.2022.1042613 (PMC9691974; doi:10.3389/fmicb.2022.1042613)
Supplement: SUPPLEMENTARY TABLE 4 — Values of gene expression used in heat map analysis. The values are a media of triplicates. [file Table_4.DOCX]

**Supplementary table S4:** **Values of gene expression used in heat map analysis. The values are a media of triplicates.**

|  | WFC-SC-014 | WFC-PK-045 | WFC-PB-047 | WFC-SP-048 | WFC-PB-051 | WFC-PB-054 | WFC-SC-C071 | WFC-SC-072 |
| --- | --- | --- | --- | --- | --- | --- | --- | --- |
| GPD1 | **7.04** | **6.33** | **1.27** | **0.08** | **15.84** | **6.32** | **16.06** | **0.70** |
| GPD2 | **59.53** | **33.21** | **44.23** | **0.20** | **1.02** | **86.82** | **22.00** | **0.04** |
| ADH1 | **27.95** | **36.78** | **13.17** | **0.14** | **0.78** | **355.17** | **39.00** | **0.11** |
| PDC1 | **0.66** | **0.05** | **1.00** | **0.06** | **1.00** | **3.20** | **153.00** | **0.81** |
| ALD6 | **105.29** | **66.34** | **3.10** | **1.00** | **0.01** | **24.69** | **883.91** | **619.13** |
| ALD4 | **2.19** | **169.04** | **0.81** | **0.08** | **0.08** | **1.55** | **327.00** | **243.25** |
| HTX1 | **2.12** | **9.43** | **0.46** | **0.19** | **0.45** | **13.96** | **41.42** | **0.01** |
| BDH1 | **375.41** | **0.44** | **0.39** | **0.02** | **0.04** | **14.35** | **105.76** | **0.04** |
| FDC1 | **58.58** | **371.28** | **500.84** | **24.83** | **87.90** | **89.34** | **149.22** | **1.78** |
| PAD1 | **0.07** | **277.15** | **315.40** | **21.40** | **15.30** | **5.21** | **32.70** | **1.79** |
| ACS2 | **4.90** | **33.83** | **0.93** | **0.01** | **0.04** | **2.83** | **18.38** | **0.84** |
| ADR1 | **2.03** | **33.92** | **1.52** | **0.07** | **0.02** | **8.20** | **696.09** | **836.46** |
